# Comprehensive Analysis of Alternative Splicing in *Digitalis purpurea* by Strand-Specific RNA-Seq

**DOI:** 10.1371/journal.pone.0106001

**Published:** 2014-08-28

**Authors:** Bin Wu, Fengmei Suo, Wanjun Lei, Lianfeng Gu

**Affiliations:** 1 Center for Bioinformatics, Institute of Medicinal Plant Development, Chinese Academy of Medical Sciences and Peking Union Medical College, Beijing, P. R. China; 2 College of Life Science, Shanxi Agricultural University, Taigu, P. R. China; 3 Institute of Integrative Genome Biology, Center for Plant Cell Biology, Department of Botany and Plant Sciences, University of California Riverside, Riverside, California, United States of America; University of Western Sydney, Australia

## Abstract

*Digitalis purpurea* (*D. purpurea*) is one of the most important medicinal plants and is well known in the treatment of heart failure because of the cardiac glycosides that are its main active compounds. However, in the absence of strand specific sequencing information, the post-transcriptional mechanism of gene regulation in *D. purpurea* thus far remains unknown. In this study, a strand-specific RNA-Seq library was constructed and sequenced using Illumina HiSeq platforms to characterize the transcriptome of *D. purpurea* with a focus on alternative splicing (AS) events and the effect of AS on protein domains. *De novo* RNA-Seq assembly resulted in 48,475 genes. Based on the assembled transcripts, we reported a list of 3,265 AS genes, including 5,408 AS events in *D. purpurea*. Interestingly, both glycosyltransferases and monooxygenase, which were involved in the biosynthesis of cardiac glycosides, are regulated by AS. A total of 2,422 AS events occurred in coding regions, and 959 AS events were located in the regions of 882 unique protein domains, which could affect protein function. This *D. purpurea* transcriptome study substantially increased the expressed sequence resource and presented a better understanding of post-transcriptional regulation to further facilitate the medicinal applications of *D. purpurea* for human health.

## Introduction

Alternative splicing (AS) is an important post-transcriptional process by which diverse transcripts are generated from one mRNA precursor. AS was first proposed by Gilbert in 1978 [1] soon after the discovery of exons and introns in the adenovirus hexon gene in 1977 [2]. AS is now recognized as a major mechanism in increasing the diversity of the transcriptome and proteomes in eukaryotic organisms. In general, AS preferentially results in alterations in the coding regions of genes rather than being randomly distributed [3], which leads to different protein variants. Protein domains are conserved protein regions with particular functions that is given according to protein sequences; protein domains can evolve, function, and exist independently from the rest of the protein chain [4], [5]. Protein isoforms with substantial changes to protein domains may play a significant role in large-scale evolution [6]. A case study demonstrated that one isoform, JAZ10.4, derived from an alternative donor site, produced a C-terminal frame-shift protein that deleted all of the Jas domain sequences [7], [8]. JAZ10.4 without the Jas domain can bind to MYC2 and other JAZ proteins but cannot bind to COI1 for degradation. Therefore, the alternative donor site can temper the JA-induced response by producing a splicing-out domain isoform that cannot be eliminated by ubiquitin E3 ligase SCFCOI1 [9].

Because AS is important in modeling protein function, thus AS events should be identified at an early stage. There are numerous experimental and bioinformatics methods for detecting AS events, including several microarray-based methods for the high-throughput detection and monitoring of AS events and their global regulation in mammals [10]–[16]. The application of an exon junction microarray [11] to animal systems is a powerful approach to detecting known splicing variants. However, the splicing junction array relies on annotated junctions and cannot detect new splicing sites. Reliance upon existing transcription has inhibited the detection of new splicing variants by a probe-based method. RNA-Seq is a recently developed high-throughput sequencing technology for transcriptome profiling and is a powerful method for identifying new splicing junctions [17]. With this technology, AS events are identified in *Drosophila* [18] and humans [19]. Genome-wide AS analysis has also been conducted in several plant species, such as rice [20], maize [21], soybean [22] and *Arabidopsis* [23].

*D. purpurea* is one of the most important medicinal plants worldwide. The main active ingredients of this plant are cardiac glycosides, which have been used to treat congestive heart failure effectively for over 200 years [24], [25]. However, few studies have been conducted on the molecular aspects of this plant. Previous studies have mainly focused on the genes involved in the biosynthesis of cardiac glycosides. For example, Kuate et al purified and characterized a malonyl-coenzyme A: 21-hydroxypregnane 21-*O*-malonyltransferase (Dp21MaT) [26]. Three groups identified genes encoding progesterone 5β-reductase (5β-POR), steroid 5β-reductase (p5βR2) and Δ^5^-3β-hydroxysteroid dehydrogenase (3βHSD) [27]–[29]. Wu et al found approximately 140 unigenes that are involved in cardiac glycoside biosynthesis and mlncRNAs that are associated with secondary metabolism and stress response [30]. However, the post-transcriptional mechanism of gene regulation in *D. purpurea* still remains largely unknown, and a comprehensive understanding of AS and its effect on protein function in *D. purpurea* is still lacking.

In this study, a comprehensive analysis of AS was performed to characterize the transcriptome of *D. purpurea* using a strand-specific RNA-Seq library. Here, we report that approximately 7% of genes are regulated by AS, including several genes involved in cardiac glycoside biosynthesis. Moreover, the functional influence of AS was also explored. The results of this study revealed that the alteration of protein domains by AS is a prevalent phenomenon in *D. purpurea*. Furthermore, the results of this study provided essential transcriptome information and will facilitate future studies on post-transcriptional regulation mechanisms in *D. purpurea*.

## Materials and Methods

### Plant material and RNA extraction

One-year-old *D. purpurea* ‘GIANT SHIRLY’ plants were grown in the experimental field of Beijing Medicinal Plant Garden of the Institute of Medicinal Plant Development, Chinese Academy of Medical Sciences & Peking Union Medical College (Beijing, China) during the natural growing seasons. At that time, the temperature ranged from 14.3°C to 30.2°C, averaging about 22.3°C. Leaves, stems, flowers and roots were collected separately from *D. purpurea* plants at the full-bloom stage on May 10, 2013 and frozen in liquid nitrogen until further use. The total RNAs of *D. purpurea* ‘GIANT SHIRLY’ were isolated using Trizol and were then treated with RNase-free DNase I to remove genomic DNA contamination. Equal quantities of total RNAs from leaves, stems, flowers, and roots, purified as described above, were then pooled for RNA-Seq library construction. The quality and quantity of the pooled RNA for the RNA-Seq sequencing library were assessed using the Agilent Technologies 2100 Bioanalyzer to have an RNA integrity number greater than 7.

### RNA-Seq library construction and Illumina sequencing

The pooled RNA of *D. purpurea* was sheared to construct two shotgun and paired-end sequencing libraries following the SMART library protocol [31]. Briefly, the first-strand cDNAs were synthesized using SMARTScribe reverse transcriptase with an oligonucleotide comprising the first Illumina adaptor with a random hexamer at the 5′ end after the first-strand cDNA synthesis. The first Illumina adaptors were added, and three non-template cytosine nucleotides were added at the 3′ end of the first-strand cDNA. Then, double-stranded cDNAs were synthesized using an oligonucleotide that contained a second Illumina adaptor with three guanine ribonucleotides. After agarose gel electrophoresis, suitable fragments were selected as templates for PCR amplification, and the final PCR products were sequenced using Illumina HiSeq 2000 as 100 nucleotide (nt) paired-end reads.

### Assembly and annotation of transcriptome

As the strand-specific RNA-Seq library for paired reads sequencing was constructed by the SMART method, RNA-Seq reads were assembled using the Trinity software [32] for *de novo* assembly with the ‘-SS_lib_type FR’ option (the first read of the fragment pair was sequenced as the sense strand, and the second read was the antisense strand) to specify the library type and to obtain a strand-specific transcript assembly. To annotate the assembled transcripts, we compared the *ab initio* assemblies with known sequences in the SWISS-PROT, NR and NT databases using BLAST with an E-value cut-off of 10^−6^. The bi-directional best hit (BBH) transcripts were categorized to search against the Kyoto Encyclopedia of Genes and Genomes (KEGG) database [33] to obtain the KO number and KEGG reference metabolic pathway.

### Gene Expression Analysis of AS

RNA-Seq has emerged as a major tool in quantitative whole-transcriptome profiling because of low background noise [34]. First, RNA-Seq reads were aligned to the assembled sequences by Bowtie2 [35]. The reads per kilobase of transcripts model per million mapped reads (RPKM) was then used to measure and normalize the gene expression, leading to the detection of the total number of transcript regions in each gene [36].

### Identification and characterization of AS

As the genome of *D. purpurea* is currently unavailable, AS is impossible to identify by a cDNA-to-genome alignment. To obtain candidate AS events, we first retained the assembled genes, which included several transcripts. The assembled sequences were then compared with each other by BLAT [37] with the following options: -tileSize = 18-oneOff = 0-minIdentity = 96-out = sim4-maxIntron = 10000. Finally, the AS events were identified according to the pair-wise comparisons of assembled transcripts to search for indels as candidate regions. Indels that were flanked by two sequentially matching sections from the pair comparison of assembled transcripts were reported as candidate AS regions. According to canonical junctions (GT-AG, GC-AG, and AT-AC splice sites) at both ends of the alternative region, the AS regions were divided into four types: intron retention, exon skipping, alternative donor sites and alternative acceptor sites [38].

### Validation by cDNA-to-genome alignment and reverse transcription PCR

To validate the method of pairwise comparison, we used *Arabidopsis*, which has an available genome sequence. All cDNA sequences based on TAIR10 annotation were then aligned to the reference genome by using Genome Mapping and Alignment Program (GMAP) [39] to detect AS events based on the cDNA-to-genome alignment and comparing the results with the predicted AS events by cDNA pairs-gapped alignment. To further validate the AS, the pooled RNA, as described for the RNA-Seq sequencing library construction, was used for reverse transcription PCR (RT-PCR) validation. Reverse transcription was conducted using 2 µg of total RNA and 200 U of SuperScript III Reverse Transcriptase in a 20 µl volume. Primers were designed by primer3 [40], and the primers used for RT-PCR are listed in Table S1. The RT-PCR reaction was performed at 65°C for 5 min, 50°C for 60 min and 70°C for 15 min. The resulting cDNA was used for RT-PCR amplification, which was performed under the following conditions: 95°C for 3 min; 45 cycles of 94°C for 30 s; 58°C for 30 s; and 72°C for 15 s. The PCR products were separated by electrophoresis using a 3% agarose gel.

### Gene ontology enrichment analysis for AS genes

To understand the functions of AS genes, all of the AS genes were mapped to the Gene Ontology (GO) database and were compared with the whole transcriptome background for GO enrichment analysis. GO enrichment analysis (*P*<0.05; hypergeometric test with Benjamini & Hochberg false discovery rate correction) was performed on all AS genes using BINGO 3.0.2 [41] according to the custom GO annotation files from the transcriptome to identify the overrepresented GO terms in AS genes.

### Detecting the functional domain affected by AS

The changes of AS in cDNA were classified into two groups: changes in the untranslated regions and changes in the coding regions. Each open reading frame (ORF) of assembled cDNAs was translated into proteins, and these variants were scanned for protein domains with HMMpfam [42]. The positional information of all the protein domains was compared with the transcript coordinates of the AS regions to determine the domains that were affected by the AS regions.

### Identification of protein domains with higher frequency of occurrences in AS

We recorded the number of every unique protein domain which belonged to constitutively splicing genes or AS genes. Then we deduced if one protein domain was higher frequency of occurrences in AS genes than constitutively splicing genes and AS genes by using Fisher’s exact test.

## Results

### RNA sequencing and *de novo* assembly

**Figure 1:**
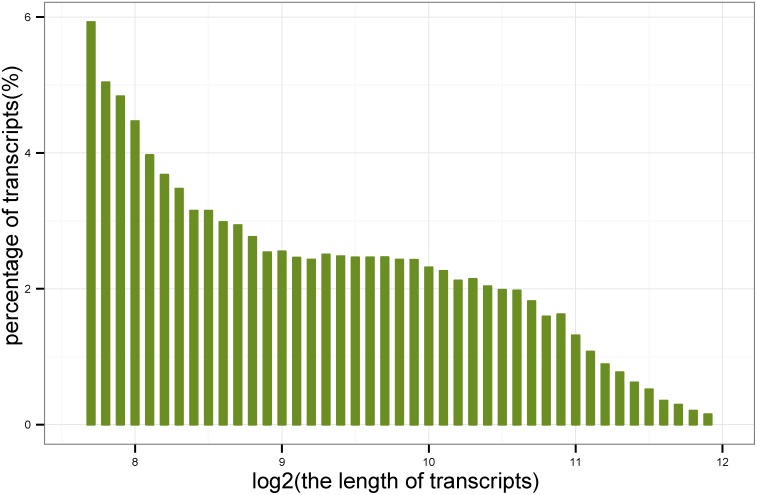
Sequence length distribution of assembled transcripts. The X-axis indicates the length range of the transcript sequences. The Y-axis indicates the percentage of transcript sequences with a certain length.

Initial *de novo* transcriptome sequencing of *D. purpurea* was constructed using a strand-specific library, which was subjected to Illumina mRNA-Seq. Illumina Solexa sequencing generated 35,201,721 paired-end reads, with a total of 7,000,403,442 nt. The transcriptome reads were then *de novo* assembled by Trinity [43] in a strand-specific way, resulting in 90,257 assembled transcripts belonging to 48,475 genes with an average length of 776 bp (**Figure 1**). In this study, 23,504 transcripts with a length greater than 1,000 nt were found, which suggested a deep sequence and advanced algorithm of the assembly software. The results in this paper can be downloaded from http://www.bioinfor.org/pub/dpu.

### Annotation of the *de novo* transcriptome

**Figure 2:**
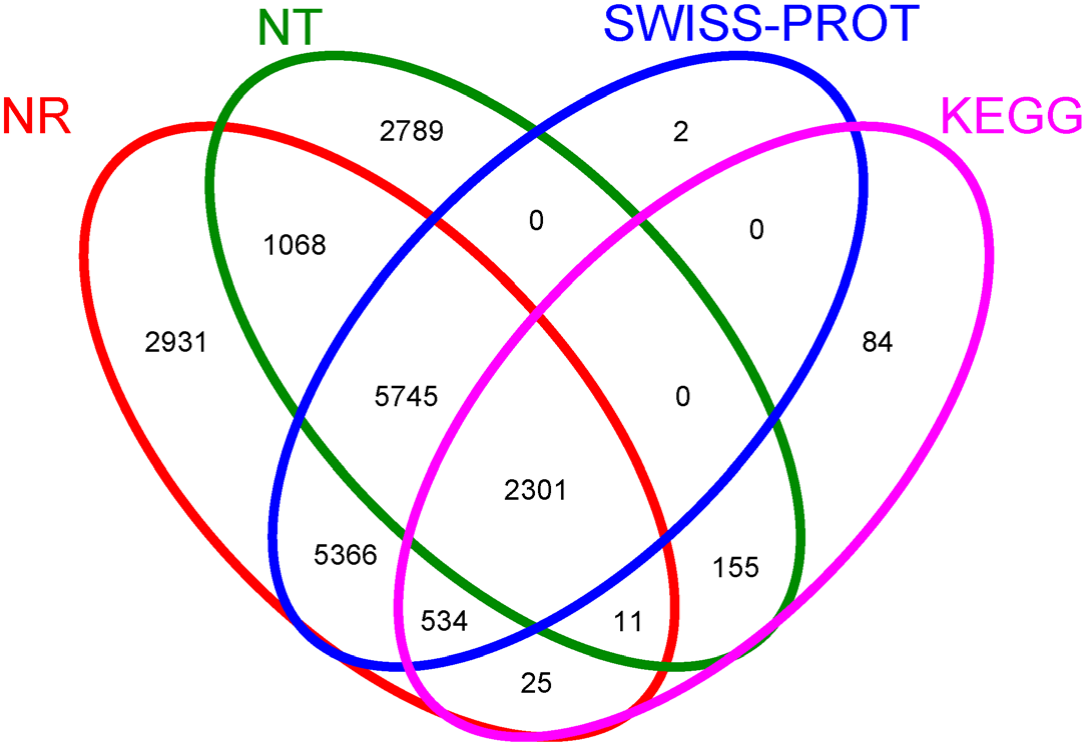
Venn diagram showing annotated genes by SP, NR, NT, and KEGG. The number of genes annotated is listed in each diagram component.

To annotate these transcripts, the assembled transcripts above were searched against the sequences in the NT database using the BLASTN algorithm (E-value<10^−6^) for functional annotations. To achieve comprehensive annotation, coding regions were extracted from the Trinity assembly transcripts using a set of utilities included in the Trinity software [43] and were searched against the SWISS-PROT and NR databases using the BLASTX algorithm (E-value<10^−6^). BLAST was used to annotate 13,948, 17,981, and 13,948 genes in the SWISS-PROT, NR and NT databases, respectively. A total of 20,927 genes were annotated, and the NR database had the largest match, followed by the SWISS-PROT and NT databases (**Figure 2**). BLAST was also performed against the KEGG database to annotate the metabolic pathways for each gene using the BBH method. A total of 3,110 genes referring to 231 pathways were identified according to the KEGG database.

### AS identification and validation

Comprehensive analysis of the assembled transcripts revealed 5,408 AS events affecting 3,265 *D. purpurea* genes based on cDNA pair-gapped alignment. To validate the feasibility of the cDNA pairs-gapped alignment method, we used *Arabidopsis* to detect AS by a cDNA-to-genome alignment, as the genome sequence of this genus was available. The results from the cDNA-to-genome alignment were then compared with the results from direct cDNA pair-gapped alignment comparisons. The method of cDNA pair-gapped alignment was consistent with the cDNA-to-genome alignment. More than 87% of the AS events based on a cDNA pair-gapped alignment overlapped completely with the method of the cDNA-to-genome alignment. Deviation was observed for multiple exon skipping (Figure S1A), which was merged into single exon skipping events (e.g., AT3G11400). We also found that an alternative position (Figure S1B) could be misclassified as intron retention (e.g., AT3G04590). The minor deviations only affected the AS classification rather than the downstream functional analysis.

**Figure 3:**
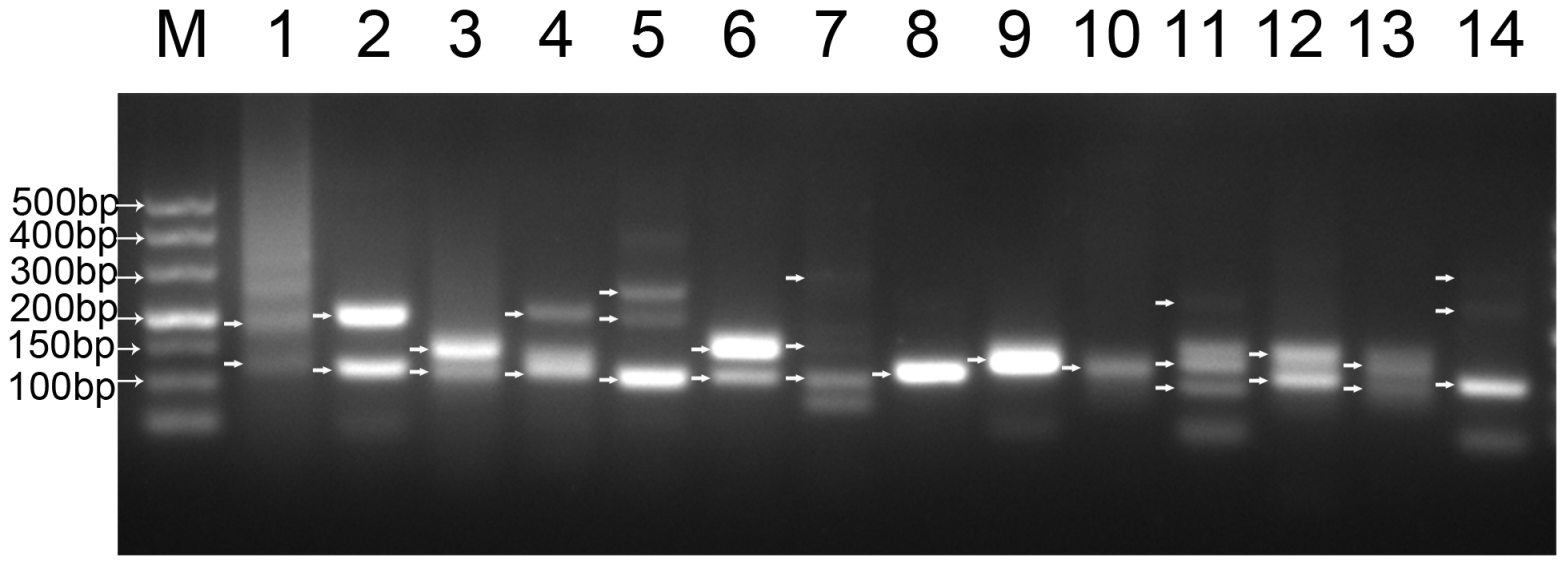
AS validation of 14 selected genes by RT-PCR. The splicing patterns of selected AS genes were analyzed using RT-PCR to validate the RNA sequencing results. Electrophoretic analysis of amplified products from RT-PCR with 3% agarose gel. M, DL500 marker, and the size of specific bands are indicated. The numbers 1–14 represent the PCR products of contig name comp 65400_c0, comp 62996_c0, comp69107_c0, comp65089_c0, comp67573_c0, comp67051_c1, comp68099_c0, comp68043_c2, comp57809_c0, comp66555_c0, comp66894_c0, comp63097_c0, comp64465_c1, and comp5406_c0, respectively.

To further validate the AS results by experimental methods, 14 genes were randomly selected and validated by RT-PCR, which showed that the sizes of the RT-PCR products were consistent with the RNA-Seq data (Figure 3). Three loci (comp68403_c2, comp57809_c0, and comp66555_c0) showed only one isoform, which was inconsistent with the RNA-seq result and might be attributed to a low abundance of the other isoforms. Among 3,265 AS genes, 2,219 genes included only one AS event. The other 32% of the AS genes included more than one AS event. For example, comp67573_c0, comp68099_c0, and comp66894_c0 included multiple bands, which suggested that more than two isoforms were produced in these loci. The primers for splicing factor SC35 (comp65400_c0) were expected to amplify two bands. We detected the expected bands in the RT-PCR result. However, we also noted that one unexpected weak band existed, which suggested that in these loci, other variants had low abundance and could not be reported by the Trinity software.

### Characterization of AS genes

Among 5,408 AS events, the percentages of intron retention, exon skipping, alternative donor sites and alternative acceptor sites were 33% (1,776/5,408), 27% (1,435/5,408), 22% (1,169/5,408) and 19% (1,028/5,408). Intron retention was the most abundant type. Up to 92% of intron retention utilized GT-AG splicing site. Only 8% of intron retention utilized GC-AG and AT-AC splicing sites. The average length for the AS region was 91 bp (Figure S2). To investigate whether AS genes had a bias regarding gene expression, the RPKM of AS genes was calculated and compared with whole transcriptome. AS genes showed higher abundance than total gene expression, and antisense transcripts showed lower abundance than sense transcripts (Figure S3).

### Biological function analysis of genes with AS events

**Figure 4:**
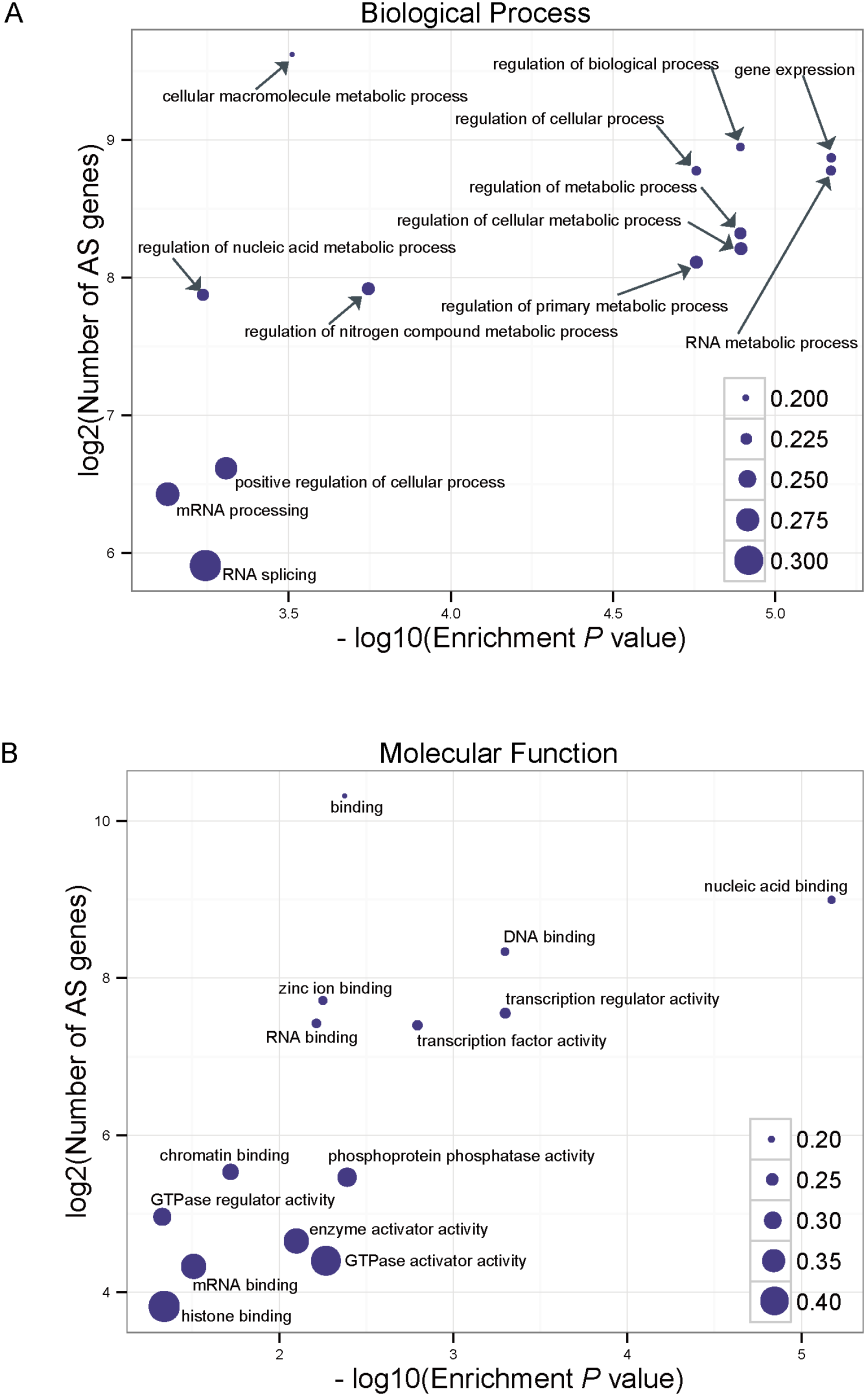
Most significant functional groups (p-value<0.05) of AS genes. The most significant functional groups (p-value<0.05) are presented graphically. The X-axis represents log10 of the enrichment P value. The Y-axis indicates the number of AS genes in log2 value. The size of each point is proportional to the percentage (AS genes associated with GO terms/All genes associated with GO terms).

GO terms were assigned to the assembled genes on the basis of the currently curated GO term annotated databases including the corresponding homologues in the SWISS-PROT database. Then, BINGO was used to identify enriched GO functional categories. A large proportion of AS genes were enriched with GO categories (biological process), such as gene expression (p-value = 6.7868E-6) and RNA splicing (p-value = 5.8775E-4) (Figure 4 A). In this study, 30% (60/194) of the RNA splicing genes were regulated by AS, which is consistent with previous reports that a splicing regulator was itself regulated by AS [38], [44]. The enrichment GO for molecular function (Figure 4B) also included transcription factor activity (1.6311E-3), RNA binding (6.2230E-3) and chromatin binding (1.9682E-2). In this study, 28% (46/162) of chromatin binding genes were regulated by AS events. Interestingly, several studies revealed that chromatin remodeling can change AS by altering transcription rates and nucleosome positioning [45], [46]. Further studies are needed to reveal the function of AS with regard to the chromatin binding genes of *D. purpurea.*

### Protein domains with higher frequency of occurrences in AS

There were 9,082 unique protein domain derived from 16,282 genes which included 13,651 constitutively spliced genes and 2,631 AS genes. In total there were 29 protein domains which had higher frequency of occurrences in AS genes (p-value<0.01, Fisher’s exact test). Phosphotransferase enzyme family (APH), RNA recognition motif (RRM), zf-C3H4 and zf-RING were four most frequently detected domain in AS genes comparing with constitutively spliced genes. RRMs are found in a variety of RNA binding proteins which implicated in regulation of splicing.

### Detecting functional domains affected by AS

There were 2,422 AS events that localized to coding regions that may alter protein function. There were 882 unique protein domains that were disturbed by these 959 AS events. Cardiac glycosides as secondary metabolites are particularly important for *D. purpurea*. Terpenoid backbone biosynthesis, steroid biosynthesis and cardenolide biosynthesis are three important steps in the biosynthetic pathway of cardiac glycosides [30]. All the genes in the cardenolide biosynthesis were indentified by searching the SWISS-PROT databases using BLAST with an E-value cut-off of 10^−6^. In this study, both UDP glycosyltransferases (including 4 AS genes) and monooxygenase (including 12 AS genes) in the cardenolide biosynthesis pathway were regulated by AS. The isoforms of randomly selected genes from 2 UDP glycosyltransferases and 2 monooxygenase were validated using RT-PCR (Figure S4 and Table S2). Among AS events in the cardenolide biosynthesis pathway, seven AS events affected protein domains. For example, intron retention on UDP glycosyltransferases genes produced two splicing transcripts with long ORFs that could be translated into complete proteins with complete UDP-glucoronosyl and UDP-glucosyl transferase domains, whereas the short transcript encoded a protein that lacked a part of the UDP-glucoronosyl and UDP-glucosyl transferase domain (Figure S5).

## Discussion

Expressed sequence tags (ESTs) and full-length cDNA (FL-cDNA) clones were excellent resources for detecting AS. Thousands of AS events were recently detected in plants by using large-scale EST/FL-cDNA-genome alignments [47]–[56] or EST-to-EST alignment [57]. However, sequencing cDNA and EST are expensive and generally not quantitative [58]. EST has also been limited because of the lack of coverage and quantify. The advantage of strand-specific RNA-Seq is the distinction of strand direction for each transcript, which is absent in the EST dataset by 454 sequencing. Although RNA-Seq has been applied to many species, strand-specific RNA sequencing has not been previously used in *D. purpurea.* In this study, transcriptome profiling was investigated using Illumina Solexa sequencing to acquire a better molecular understanding of post-transcriptional regulation in *D. purpurea*. *De novo* RNA-Seq assembly resulted in 90,257 transcripts belonging to 48,475 genes. This dataset had advantages compared with the previous EST dataset because this study investigated the transcriptome profiles of *D. purpurea* and provided the first comprehensive insight into post-transcriptional regulation by using strand-specific RNA-Seq. Strand direction is important for AS identification because natural antisense transcripts (NAT) are also ubiquitous. Without strand direction, AS events are difficult to distinguish from NAT events. Comprehensive transcriptome analysis revealed 3,265 genes with 5,408 AS events. To assess the results of RNA-seq, RT-PCR was used to examine the splicing profiles, which revealed that the splicing events based on RNA sequencing were reliable. More than 7% of genes in *D. purpurea* were involved in AS events. The percentage of AS was underestimated because the depth of RNA-seq is insufficient to cover all AS events. Transcripts with low abundance were not be assembled by the Trinity software [32]. The number of AS genes will increase with deeper sequencing, tissue, and development stage.

In this study, AS events were further classified into intron retention, exon skipping, alternate acceptor and alternate donor sites according to the consensus intron border (GT-AG, GC-AG, AT-AC), and thus, non-canonical splicing sites (junctions with intron edges other than GT-AG, GC-AG, or AT-AC) were not considered for AS classification. As the percentage of genes with non-canonical splicing sites is low, non-canonical splicing can be ignored.

According to previous report AS expression is regulated in specific tissues and developmental stages by reciprocity between different trans splicing factors and complex cis regulatory elements [59]. SR (Serine/arginine-rich) proteins and hnRNPs are two currently well-known trans splicing factors that can either facilitate or restrain spliceosome assembly [38]. In this study, GO enrichment analysis of the AS genes showed that the GO terms RNA splicing, chromatin binding and transcription factor were significantly enriched for GO functional categories. A previous study reported that splicing, chromatin modifications and transcription are highly integrated and influence each other [60]. Transcription factors can effect AS by influencing the rate of RNA polymerase II elongation [61].

Recent studies by both experimental and bioinformatics methods have shown a large number of alternative spliced variants in plants. However, investigating the functional effect of AS has remained challenging. Hence, the elucidation of functional diversity is critical for understanding the importance of AS. In total, 45% of AS events were localized to the coding region, and 959 AS events were predicted to affect protein domains. The length of the AS region was analyzed to detect whether the length of the AS events would be an integer multiple of three. An integral number of codons caused by AS events will retain the same translation frame, but a shift in the translation frame will result if the AS regions are not an integer multiple of three. Among 2,422 AS events localized to coding regions, 1,277 AS events are predicted to retain their translational frame, with the remaining 1,145 AS undergoing a translational frame-shift. A recent genome-wide study showed that splicing variants could initiate the translation step [62], [63], suggesting that these alternative protein isoforms might have important functions. Thus, investigating the ability of translational frame-shift isoforms to initiate the translation step is of interest in future studies.

## Conclusions

The transcriptome results in this study provided comprehensive assembled sequence information on *D. purpurea* for future research. Furthermore, this study provided comprehensive AS information that will facilitate our understanding of the important post-transcription regulation mechanisms in *D. purpurea*. Finally, the effect of AS on protein domains was investigated, and the results suggested that AS might have a general role in modeling protein structure and function.

## Supporting Information

Figure S1 **Visualization the deviation of cDNA pair-gapped alignment and the cDNA-to-genome alignment.**
(TIF)

Figure S2 **Length distribution of the AS region.** The X-axis indicates log2 of the length of the AS region. The Y-axis indicates the percentage of AS events with a specific length.
(TIF)

Figure S3 **Plotting distribution of log-transformed RPKM values for AS genes and antisense transcripts.** The X-axis displays log2 of the RPKM value. The Y-axis indicates the percentage of genes with a given RPKM value.
(TIF)

Figure S4 **Validation of the AS for UDP glycosyltransferases and monooxygenases.** The splicing patterns of two UDP glycosyltransferases and two monooxygenase were validated by RT-PCR. Electrophoresis analysis of amplified products from RT-PCR with 3% agarose gel. M, DL500 marker, size of major bands were indicated.
(TIF)

Figure S5 **Visualization of intron retention on UDP-glucoronosyl and UDP-glucosyl transferase domain.**
(TIF)

Table S1 **Primers of 14 genes for AS validation.**
(DOC)

Table S2 **Primers for AS validation of UDP glycosyltransferases and monooxygenase.**
(DOC)
