# Supplementary figures and images for: Comprehensive Analysis of Alternative Splicing in Digitalis purpurea by Strand-Specific RNA-Seq
Source: PLoS One. 2014 Aug 28;9(8):e106001. doi: 10.1371/journal.pone.0106001 (PMC4148352; doi:10.1371/journal.pone.0106001)

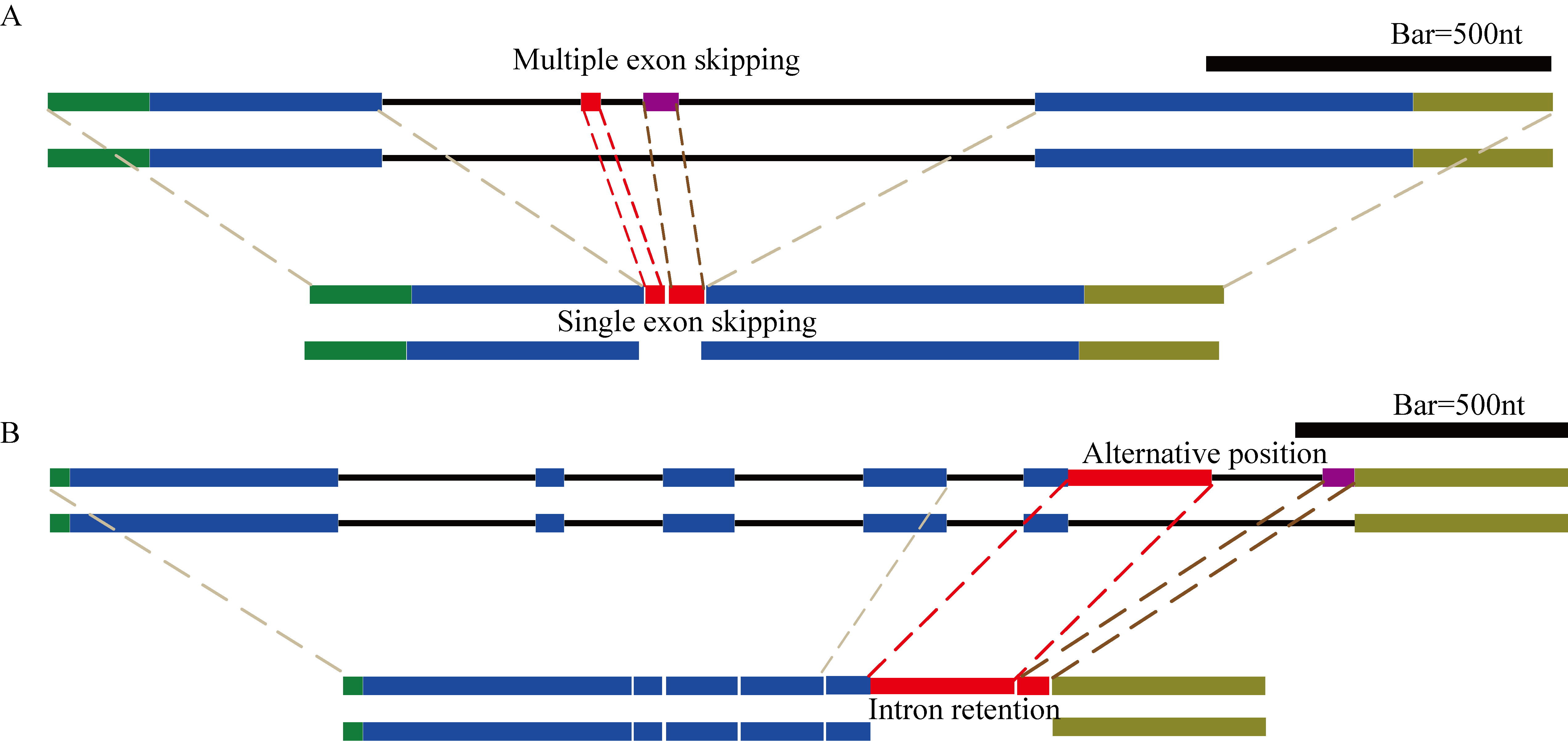

Supplement: Figure S1 — Visualization the deviation of cDNA pair-gapped alignment and the cDNA-to-genome alignment. (TIF) [file pone.0106001.s001.tif]

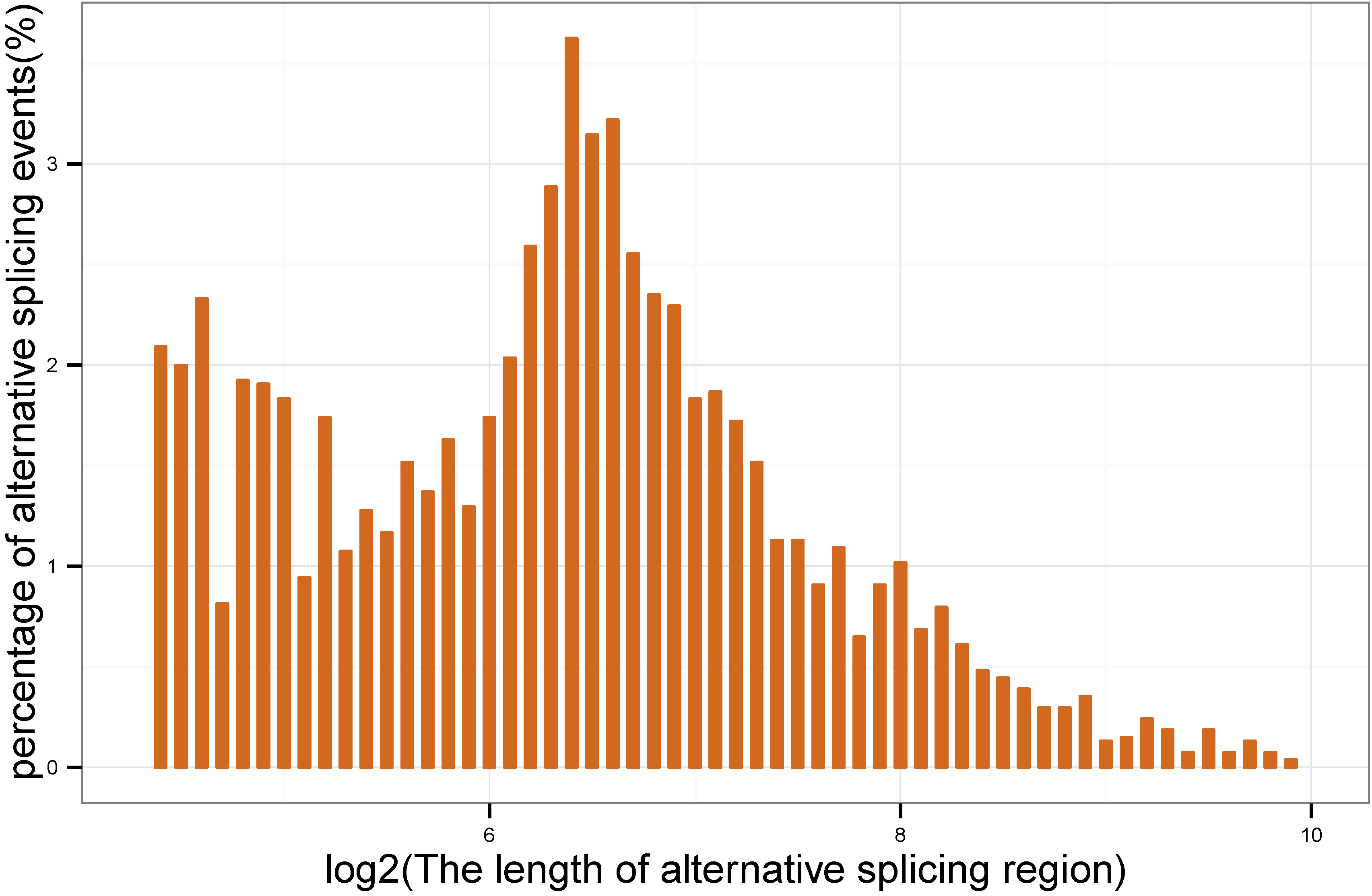

Supplement: Figure S2 — Length distribution of the AS region. The X-axis indicates log2 of the length of the AS region. The Y-axis indicates the percentage of AS events with a specific length. (TIF) [file pone.0106001.s002.tif]

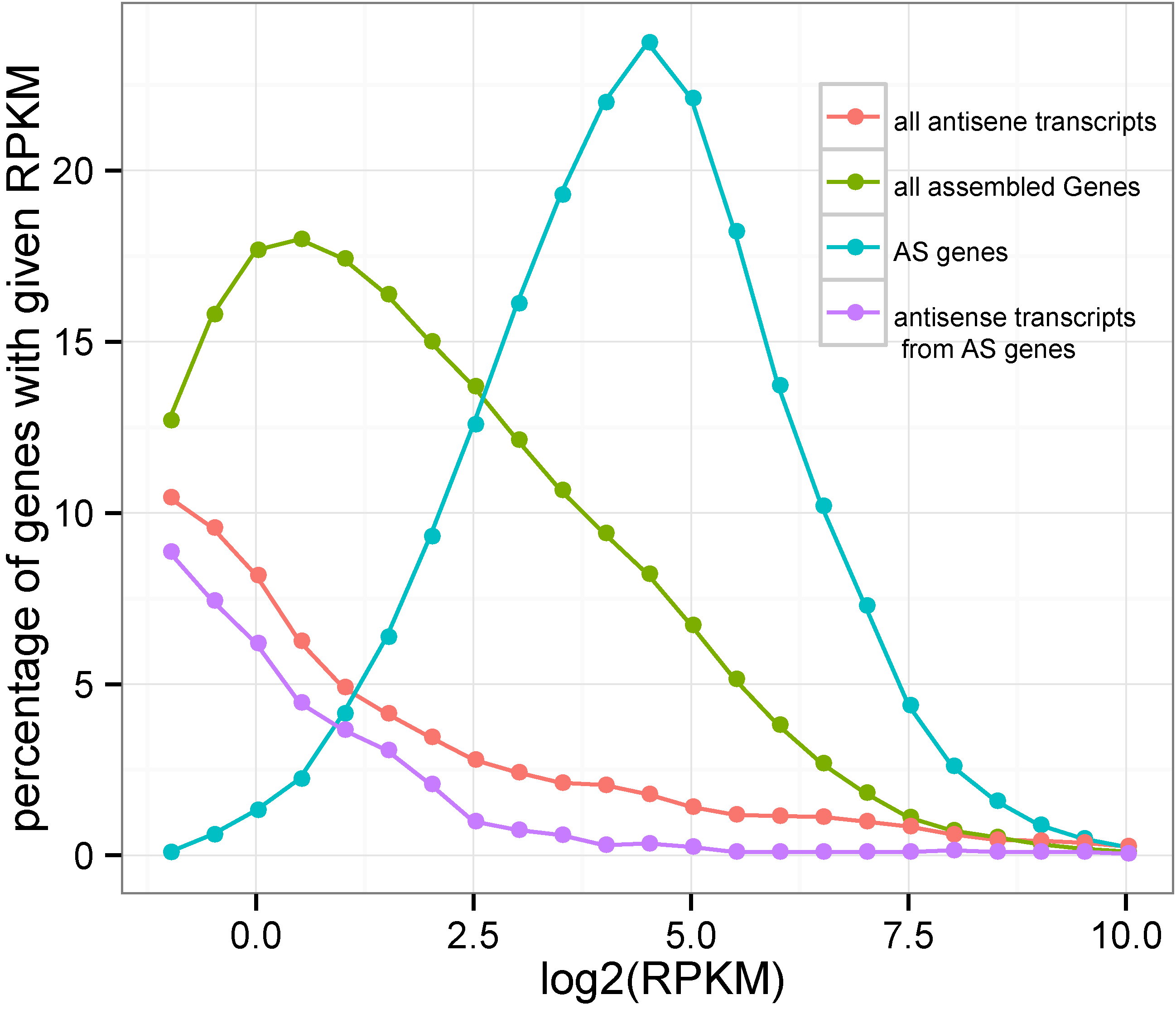

Supplement: Figure S3 — Plotting distribution of log-transformed RPKM values for AS genes and antisense transcripts. The X-axis displays log2 of the RPKM value. The Y-axis indicates the percentage of genes with a given RPKM value. (TIF) [file pone.0106001.s003.tif]

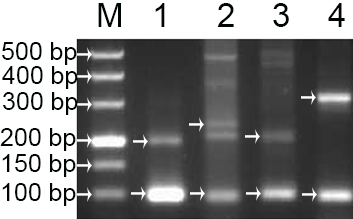

Supplement: Figure S4 — Validation of the AS for UDP glycosyltransferases and monooxygenases. The splicing patterns of two UDP glycosyltransferases and two monooxygenase were validated by RT-PCR. Electrophoresis analysis of amplified products from RT-PCR with 3% agarose gel. M, DL500 marker, size of major bands were indicated. (TIF) [file pone.0106001.s004.tif]

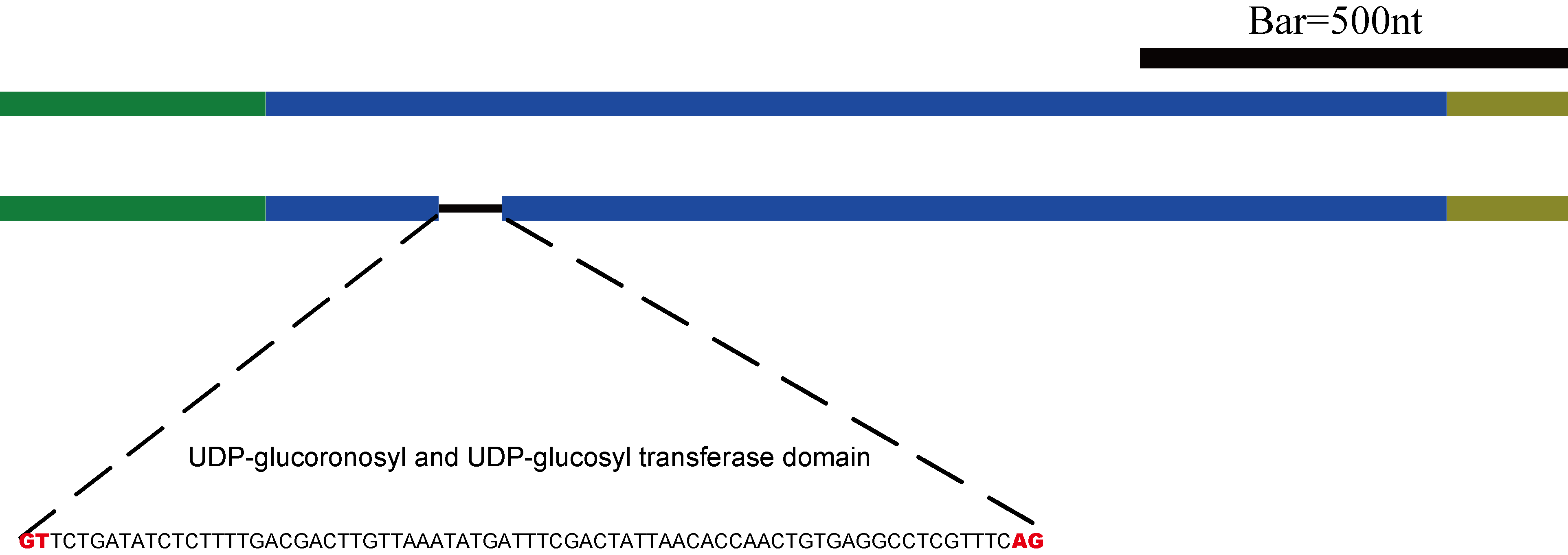

Supplement: Figure S5 — Visualization of intron retention on UDP-glucoronosyl and UDP-glucosyl transferase domain. (TIF) [file pone.0106001.s005.tif]
