# Supplementary material for: Comprehensive Analysis of Alternative Splicing in Digitalis purpurea by Strand-Specific RNA-Seq
Source: PLoS One. 2014 Aug 28;9(8):e106001. doi: 10.1371/journal.pone.0106001 (PMC4148352; doi:10.1371/journal.pone.0106001)
Supplement: Table S1 — Primers of 14 genes for AS validation. (DOC) [file pone.0106001.s006.doc]

**Table S1. Primers of 14 genes for alternative splicing validation**

| **Contig Name** | **Primer sequences (5'-3')** | **PCR products size (bp)** | **Annotation** |
| --- | --- | --- | --- |
| comp65400_c0 | Forward: GAGTTATGGAACCTGTTTCCAA  Reverse: GCTTCTGGATCTCCCTCTGGACA | 184，122 | Splicing factor SC35 |
| comp62996_c0 | Forward: CATAGCGGAGATAACCGACATAG  Reverse: CAATCTTTCATCCACTCCACCTC | 203，119 | NA |
| comp69107_c0 | Forward: GTCTTGTACCTTCGGCTTCG  Reverse: GCATTCTCGGGTCTTGCAAA | 146，115 | Eukaryotic translation initiation factor 3 |
| comp65089_c0 | Forward: ATCAACTCCATTCAACCAATACG  Reverse: TTGGGGATTTTGTGCTGCTG | 213，114 | Retrotransposon gag protein |
| comp67573_c0 | Forward: GCAAATCTGCACTCTTGTACAGA  Reverse: ACCACGAAAATTCACAGAAGCA | 260，202，105 | Serine/threonine-protein kinase SRK2B |
| comp67051_c1 | Forward: GCTAAGCCCAAGGCGGC  Reverse: GTCTTTGACTTCGCCGGAG | 152，110 | Histone H1.2 |
| comp68099_c0 | Forward: GCCGACACAGCAGAGATGAA  Reverse: AAATCAGCTCCACTCACTGC | 310， 158，108 | Casein kinase I isoform delta |
| comp68403_c2 | Forward: CGTAAGGCAAATAGTAAGTCACAT  Reverse: TGGCACATAATATCATCTTGGCT | 232，114 | Phospholipid-transporting ATPase 3 |
| comp57809_c0 | Forward: GGCTTAACTGCGAACCAACA  Reverse: GTCAGTTTTAGGAATGCGTTGC | 137，112 | Protein TWIN LOV 1 |
| comp66555_c0 | Forward: GAAGAAACACTCAGCTCCCGAA  Reverse: TTGTCATGAATTTCCTTGTCCTG | 127，95 | 40S ribosomal protein S24-2 |
| comp66894_c0 | Forward: AGTTCGCTGTGTTGCTTCAA  Reverse: TCACTGCAAAGCTACACACTC | 246， 134，99 | Protein SIP5 |
| comp63097_c0 | Forward: TGGTCCAGTTGTATTGGGCT  Reverse: ACACAAGGAATAAAACGCTAACTT | 140，114 | NA |
| comp64465_c1 | Forward: TGTTAAGGAGAAAGGTAGCCTCA  Reverse: CTCGTCAGTAAATTCATGGTCGT | 135，105 | Protein TIFY 10B |
| comp65406_c0 | Forward: CCACCGATGACAAGTCCCT  Reverse: AGTCACAAATCCGAATCCCC | 377，247，113 | Glycine-rich RNA-binding protein blt801 |
