# Supplementary material for: Comprehensive Analysis of Alternative Splicing in Digitalis purpurea by Strand-Specific RNA-Seq
Source: PLoS One. 2014 Aug 28;9(8):e106001. doi: 10.1371/journal.pone.0106001 (PMC4148352; doi:10.1371/journal.pone.0106001)
Supplement: Table S2 — Primers for AS validation of UDP glycosyltransferases and monooxygenase. (DOC) [file pone.0106001.s007.doc]

**Table S2. Primers of UDP-glycosyltransferase and Monooxygenase genes for alternative splicing validation**

| **Contig Name** | **Primer sequences (5'-3')** | **PCR products size (bp)** | **Annotation** |
| --- | --- | --- | --- |
| comp50224_c0 | Forward: TCCGAACCCGAAAAACTACC  Reverse: GCCACCTCACCATTGAACTC | 120，193 | UDP-glycosyltransferase |
| comp63880_c0 | Forward: CTCTAAGTTACCACGTCCAGCTC  Reverse: TGGACTCAACTCCTGGAAGG | 120，261 | UDP-glycosyltransferase |
| comp65534_c0 | Forward: AGCACGCCAGCTGGACTAT  Reverse: TCACTCCTCCGTCAAACAAA | 120，221 | Monooxygenase |
| comp62694_c0 | Forward: CAGTTGCTAATGCTGGTGGA  Reverse: TGGTTTGTCAGTTAAAGTTTTTGTC | 120，326 | Monooxygenase |
